# Second malignancies after breast cancer: the impact of different treatment modalities

**DOI:** 10.1038/sj.bjc.6604241

**Published:** 2008-02-12

**Authors:** Y M Kirova, Y De Rycke, L Gambotti, J-Y Pierga, B Asselain, A Fourquet

**Affiliations:** 1 Department of Radiation Oncology, Institut Curie, Paris, France; 2 Department of Biostatistics, Institut Curie, Paris, France; 3 Department of Medical Oncology, Institut Curie, Paris, France

**Keywords:** breast cancer, second malignancies, second malignant neoplasms, radiotherapy, chemotherapy, hormone therapy, long-term risk

## Abstract

Treatment for non-metastatic breast cancer (BC) may be the cause of second malignancies in long-term survivors. Our aim was to investigate whether survivors present a higher risk of malignancy than the general population according to treatment received. We analysed data for 16 705 BC survivors treated at the Curie Institute (1981–1997) by either chemotherapy (various regimens), radiotherapy (high-energy photons from a ^60^Co unit or linear accelerator) and/or hormone therapy (2–5 years of tamoxifen). We calculated age-standardized incidence ratios (SIRs) for each malignancy, using data for the general French population from five regional registries. At a median follow-up 10.5 years, 709 patients had developed a second malignancy. The greatest increases in risk were for leukaemia (SIR: 2.07 (1.52–2.75)), ovarian cancer (SIR: 1.6 (1.27–2.04)) and gynaecological (cervical/endometrial) cancer (SIR: 1.6 (1.34–1.89); *P*<0.0001). The SIR for gastrointestinal cancer, the most common malignancy, was 0.82 (0.70–0.95; *P*<0.007). The increase in leukaemia was most strongly related to chemotherapy and that in gynaecological cancers to hormone therapy. Radiotherapy alone also had a significant, although lesser, effect on leukaemia and gynaecological cancer incidence. The increased risk of sarcomas and lung cancer was attributed to radiotherapy. No increased risk was observed for malignant melanoma, lymphoma, genitourinary, thyroid or head and neck cancer. There is a significantly increased risk of several kinds of second malignancy in women treated for BC, compared with the general population. This increase may be related to adjuvant treatment in some cases. However, the absolute risk is small.

The overall survival rate of patients with early advanced breast cancer (BC) has increased over the years largely because adjuvant therapy, whether chemotherapy, radiotherapy or hormone therapy, has helped prevent local and distant failures (Fox, 1979; Jones and Raghavan, 1993; EBCTCG, 2005). Second malignancies that occur in long-term survivors may be due to sporadic cancers that would have occurred anyway, environmental or genetic factors (Klijn *et al*, 1997; Schrag *et al*, 1997; Turner *et al*, 1999; Meijers-Heijboer *et al*, 2000; Pierce *et al*, 2000, 2003; Stoppa-Lyonnet *et al*, 2000; Galper *et al*, 2002; Kauff *et al*, 2002; Pierce, 2002; Robson, 2002; Seynaeve *et al*, 2004; Kirova *et al*, 2005a, 2005b, 2006a; Laki *et al*, 2007), or BC treatment (Neugut *et al*, 1993; Inskip *et al*, 1994; Ahsan and Neugut, 1998; Karlsson *et al*, 1998; Kirova *et al*, 1998, 2005a, 2005b, 2007; Obedian *et al*, 2000; Rubino *et al*, 2000; Scholl *et al*, 2001; Shousha *et al* 2001; Yap *et al*, 2002, 2005; Deutsch *et al*, 2003; Zablotska and Neugut, 2003; Zablotska *et al*, 2005; Mellemkjaer *et al*, 2006)

The aim of this study was to estimate the risk of a second malignancy after adjuvant treatment for BC in a homogeneous cohort of patients from a single institution. The observed incidence of second malignancies in these BC patients was compared with the expected age-adjusted number of new cases in the general population of French women as given by data from five regional registries (Remontet *et al*, 2003).

## PATIENTS AND METHODS

We analysed data for 16 705 consecutive patients with non-metastatic BC who were treated at the Institut Curie between 1981 and 1997. The data, including treatments, were entered prospectively into the Institute's BC database set up in 1981 (Salmon *et al*, 1997). Chemotherapy regimens in adjuvant and neoadjuvant setting varied over time based on CMF (cyclophosphamide, methotrexate and 5-fluorouracile), FAC (5-fluorouracile, adriamycine and cyclophosphamide) or FEC (epirubicin). All patients received alkylating agents and the majority received anthracyclins. Hormonal therapy consisted mostly of 2–5 years of administration of tamoxifen. Patients who underwent radiotherapy received high-energy photons produced by a ^60^Co unit or linear accelerator, as previously described, either as sole treatment or pre- or post surgery (Fourquet *et al*, 1991; Campana *et al*, 2005; Kirova *et al*, 2006b). Follow-up included a six-monthly clinical examination and a once-yearly mammogram for 5 years, and then a once-yearly clinical examination and a unilateral or bilateral mammogram for the lifetime of every patient. All follow-up data were entered into the database. At 5 years, 5% of patients were lost to follow-up and at 10 years, 8% were lost to follow-up.

We recorded clinical and primary tumour variables, radiation history and irradiation fields, for all patients with histologically confirmed second malignancies. Second malignancies included all first cancers occurring after treatment of the primary BC, but excluded contralateral BC.

### Statistical analysis

We first calculated Kaplan–Meier cumulative incidence and the 10-year risk of developing each type of second malignancy (Kaplan and Meier, 1958). The observed crude incidence rates in the entire patient population (cases per 100 000 person-years) were then compared with the expected incidence in the general population of French women as given by age-standardized data from five regional registries (Remontet *et al*, 2003), and a standardized incidence ratio (SIR) was calculated for each malignancy. We then calculated the SIRs for the highest-risk malignancies according to the adjuvant treatment the patients had received to study the impact of treatment on risk. The Poisson regression model was used to adjust the analysis. The data were analysed using ‘S Plus 6.2, Insightful Corp.’ software.

## RESULTS

Median follow-up was 10.5 years (range 0.2–24 years). Median patient age at the time of BC diagnosis was 56.2 years. Of the total population of 16 705 patients, 13 472 (80.6%) received radiation therapy, 2347 (17.4%) underwent mastectomy followed by radiotherapy, 8596 (63.8%) lumpectomy then radiotherapy, and 2529 (18.8%) were treated by radiotherapy alone. A total of 4528 patients (27.1%) received chemotherapy (14.3% chemotherapy alone; 12.8% chemotherapy plus hormone therapy) and 16.5% received hormone therapy alone. Overall, 9414 patients (56.4%) did not receive any systemic adjuvant therapy. The number of patients receiving different treatment combinations is given in Table 1.

By 10.5 years of median follow-up, 709 patients had developed a second malignancy. Table 2 gives the cumulative incidence of second malignancies 10.5 years after BC in the study population by decreasing order of incidence. Gastrointestinal (GI) cancer was the most common cancer, followed by gynaecological cancer (cervical and endometrial) and ovarian cancer. Table 3 compares the observed crude incidence in patients and the incidence in the general population of French women. Of all the malignancies, only leukaemia, ovarian and other gynaecological cancers (cervical and endometrial), and GI tumours, showed a significantly higher incidence in patients than in the general population. Among the 74 patients with histologically confirmed primary ovarian cancer, 13 underwent genetic testing because they presented a familial history of BC or ovarian cancer and, of these 13 patients, 10 were carriers of *BRCA* mutations (9 of *BRCA1*, 1 of *BRCA2*).

The extent to which the different treatments constituted risk factors for a second malignancy is shown in Table 4. Chemotherapy was the most important risk factor for leukaemia and highly significantly increased the risk of this disease. Radiotherapy was a much less significant risk factor. Both hormone treatment and radiotherapy were significant risk factors for gynaecological cancers. The SIR of ovarian cancer was threefold higher in patients who had received radiotherapy plus chemotherapy than in patients receiving no adjuvant therapy. The combination treatment was a highly significant risk factor. Chemotherapy alone had no significant effect maybe because of the small number of events and lack of statistical power. We found no relationship between GI tumours and BC treatment (not shown).

## DISCUSSION

To our knowledge, this is the largest retrospective study from a single institution on second malignancies and one of the first to attempt to relate the incidence and risk of a second malignancy in patients with non-metastatic BC to the expected number of cases in the general population of women of the same age, after stratifying patients by treatment received (Rubino *et al*, 2000). Patients treated for BC showed increased risk of leukaemia, ovarian cancer, and gynaecological cancers, and a slightly enhanced risk of GI cancers, in addition to the well-known risk of developing sarcomas (Kirova *et al*, 2005b) and lung cancer after radiation therapy (Kirova *et al*, 2007). The increase in leukaemia was most strongly related to chemotherapy (alkylating agents) and that in gynaecological cancers to hormone therapy (the main treatment was tamoxifen). Radiation therapy alone also had a significant, but lesser, effect found only in comparison with the general population (Rubino *et al*, 2000).

There was no difference between irradiated and non-irradiated patients with regard to leukaemia risk (Kirova *et al*, 2007), but there was a significant difference between our patients and the general population. Such a difference has already been noted and has been related to the use of adjuvant chemotherapy (7, 13, 19, 34; Rubino *et al*, 2000). At the Institut Gustave Roussy, the overall SIR for leukaemia was 3.1 (95% confidence interval (CI): 1.7–5.0) in 4416 BC patients and 2.1 (95% CI: 1.1–3.5) in the subpopulation of 416 patients receiving chemotherapy (Rubino *et al*, 2000).

Our observation of an increased risk of ovarian cancer confirms previous findings (Easton *et al*, 1993; Breast Cancer Linkage Consortium, 1997; Fisher *et al*, 1998; Chappuis *et al*, 2000; Haber, 2002; Haffty *et al*, 2002; Kauff *et al*, 2002; Pierce *et al*, 2003; Blamey *et al*, 2004) and suggests that these patients may have a familial predisposition to BC and ovarian cancer. Although we tested 13 of 74 patients with ovarian cancer for *BRCA1* or *BRCA2* mutations and found a mutation in 10 of the 13 patients with familial cancer, this result is not representative of the whole population of patients. We included patients as from 1981, but only began genetic testing in the early nineties. The increased risk of endometrial cancer might be due to tamoxifen use, as shown by others (Ewertz and Mouridsen, 1985; Brenner *et al*, 1993; Volk and Pompe-Kirn, 1997). Confirmation of this would need distinguishing different types of hormone therapy (anti-estrogens, anti-aromatase) from surgical hysterectomy and radiation-induced castration.

No relationship between GI cancers and different treatment modalities was observed. This and our previous study did not find increased incidence of oesophageal cancers, related to the radiation treatment (Kirova *et al*, 2007).

A major strength of our study is the large volume of individual patient data from a single institution. This differentiates it from epidemiological studies that lack individual data on patient treatment and from most single-institution series that are much smaller. However, despite the large number of patients and long follow-up (10.5 years), the incidence of second malignancies may nevertheless remain underestimated because of the long latency period of some tumours.

In conclusion, this study has confirmed an increased risk of second malignancies in women treated for BC, compared with the general population. This increase may be related to adjuvant treatment in some cases. However, the absolute risk is small and the influence of other predisposing factors, such as for instance family history of cancer and history of smoking, will need to be investigated in a prospective study, preferably with a long enough follow-up to exclude other late complications.

## Figures and Tables

**Table 1:** Distribution of patients by treatment

	**Number of patients (%)**		
**Systemic treatment**	**No radiotherapy**	**Radiotherapy**	**Total**
None	2371	7043	9414 (56.4)
Chemotherapy (CT)	169	2221	2390 (14.3)
Hormone therapy (HT)	509	2254	2763 (12.8)
CT and HT	185	1953	2138 (16.5)
Total	3234 (19.4)	13 472 (80.6)	16 705 (100)

**Table 2:** Ten-year cumulative incidence of second malignancy (Kaplan–Meier estimates)

	***n***	**(%)**	**95% CI**
Gastrointestinal	182	11.5	9.5–13.4
Gynaecological^a^	132	8.6	6.9–10.2
Ovary	74	5.0	3.8–6.3
Lung	58	3.7	2.6–4.9
Leukaemia	47	3.3	2.3–4.3
Melanoma	37	2.8	1.8–3.8
Lymphoma	41	2.6	1.7–3.5
Genitourinary	41	2.2	1.4–3.0
Sarcoma	34	2.1	1.3–3.0
Others	25	1.6	0.8–2.3
Thyroid	20	1.4	0.7–2.1
Head and neck	18	1.1	0.5–1.6
Total	709	45.0	41.2–48.7

Abbreviation: CI=confidence interval.

a Cervical and endometrial.

**Table 3:** Crude cancer incidence rates in patients and in the general population of French women (by decreasing order of SIR)

	**Patients**	**General population**			
**Second malignancy**	**Crude incidence rates 100 000 PY**		**SIR**	**95% CI**	***P***
Leukaemia	30.4	8.7	2.07	1.52–2.75	<0.0001
Ovarian	47.6	14.9	1.6	1.27–2.04	<0.0001
Gynaecological	86.0	28.0	1.6	1.34–1.89	<0.0001
Lung	37.5	15.2	1.2	0.91–1.55	0.16
Malignant melanoma	23.9	13.8	1.07	0.76–1.48	0.67
Lymphoma	26.9	16.6	0.95	0.68–1.29	0.76
Genitourinary	26.6	15.8	0.89	0.64–1.20	0.44
Thyroid	12.9	9.6	0.87	0.53–1.35	0.54
Gastrointestinal	119.1	15.9	0.82	0.70–0.95	0.007
Head and neck	11.6	9.1	0.64	0.38–1.01	0.06
Sarcoma	22.3	NA^a^	—	—	—

Abbreviations: CI=confidence interval; PY=person-years; SIR=standardized incidence ratio.

a NA, not available by age for the general population of French women.

**Table 4:** Standard SIR for high-risk malignancies according to adjuvant treatment

	**SIR**	**95% CI**	***P***
*Leukaemia*			
General population	1		
No adjuvant therapy	0.99	(0.14; 7.05)	0.99
RT alone	1.86	(1.26; 2.73)	0.0016
CT alone	13.31	(4.99; 35.46)	<0.0001
RT+CT	3.63	(2.06; 6.39)	<0.0001
			
*Gynaecological cancer*			
General population	1		
No adjuvant therapy	0.89	(0.13; 6.31)	0.91
RT alone	1.52	(1.20; 1.92)	0.0006
HT alone	2.90	(1.45; 5.80)	0.0026
RT+HT	2.39	(1.67; 3.41)	<0.0001
			
*Ovarian cancer*			
General population	1		
No adjuvant therapy	0.80	(0.11; 5.70)	0.82
RT alone	1.46	(1.07; 1.98)	0.01
CT alone	1.38	(0.19; 9.77)	0.75
RT+CT	3.06	(2.07; 4.53)	<0.0001

Abbreviations: CI=confidence interval; CT=chemotherapy; HT=hormonal therapy; RT=radiotherapy, SIR=standardized incidence ratio.
